# Waste Originating from the Cleaning of Flue Gases from the Combustion of Industrial Wastes as a Lime Partial Replacement in Autoclaved Aerated Concrete

**DOI:** 10.3390/ma15072576

**Published:** 2022-03-31

**Authors:** Agnieszka Różycka, Łukasz Kotwica

**Affiliations:** Department of Building Materials Technology, Faculty of Materials Science and Ceramics, AGH University of Science and Technology, 30-059 Kraków, Poland; lkotwica@agh.edu.pl

**Keywords:** autoclaved aerated concrete, calcareous waste, CO_2_ emission, calcium silicate hydrates, 1.1 nm tobermorite

## Abstract

This paper aims to study the suitability of partial replacement of lime by waste originating from the cleaning of flue gases from the combustion of industrial wastes in the production of autoclaved aerated concrete (AAC). The compressive strength, bulk density, pore structure, phase composition, and microstructure of hydration products of the AAC were analyzed. According to the results, the addition of the waste can effectively enhance the mechanical properties of AAC due to the differences in morphology of hydration product—1.1 nm tobermorite and related dense microstructure. The pore size distribution was significantly influenced by waste addition, which was one of the main reasons for the increase in thermal conductivity. The XRD and SEM results showed that foreign ions introduced with the wastes affect the synthesis of 1.1 nm tobermorite. Moreover, it was shown that waste containing a high content of CaO can be used as lime replacement, which allows reducing CO_2_ emissions during the AAC production process.

## 1. Introduction

Autoclaved aerated concrete (AAC) is attracting considerable interest and large industrial demand. AAC is currently one of the most popular building materials used in residential and industrial construction in Poland [1]. The porous microstructure of concrete and the matrix formed mainly by hydrated calcium silicates (poorly crystalline C–S–H phase and 1.1 nm tobermorite), which are responsible, inter alia, for the mechanical strength, result in aerated concrete combining the advantages of construction and insulation material [2]. The basic technological operations during AAC production include [2] preparation of raw materials (cement, lime, quartz sand), mixing of raw materials in a water suspension with the addition of aluminum powder (an aerating agent), growth of mass (related to the evolution of hydrogen as a result of the reaction of aluminum powder with Ca(OH)_2_), precuring process (obtaining a hardness sufficient for further technological operations, related to cutting into blocks), cutting hardened concrete mass into blocks, autoclaving (achieving the final strength of the products), cooling, and storage. 

Current trends in the construction and the production of building materials aim to reduce the consumption of energy and natural resources in all production processes. Although AAC production technology is characterized by relatively low consumption of raw materials and energy, research is being carried out to replace traditionally used raw materials with industrial wastes [3,4,5,6,7,8,9,10,11,12,13,14,15,16,17,18,19,20,21].

According to literature data, utilization of industrial wastes in AAC production allows modifying the properties of AAC [14,15,16]. Generally, wastes are considered as a partial replacement for cement or quartz sand. MSWI bottom ash [10], perlite waste [9] rice husk ash [3], iron tailings [6], graphite tailings [16], and blast furnace slag [21] have been considered as siliceous resources. Sodium carbonate activated slag [17], bottom ash [18], granulated slag, and metakaolin [14] have been used as cement replacement in autoclaved aerated concrete. However, although many studies [3,4,5,6,7,8,9,10,11,12,13,14,15,16,17,18,19,20,21] in recent years have concentrated on the recycling of different types of byproducts or residues in AAC technology, fewer reports [11,19] are found about using calcareous wastes to replace lime in the production of AAC. Hauser et al. [19] studied the influence of fly ash from the cellulose industry used as calcareous material on the AAC properties. The results showed that lime–sulfate ash had a positive effect on the mechanical properties of AAC. However, a higher proportion of fly ash caused a delay in the formation of hydrated calcium silicate phases. The addition of waste to concrete resulted in a decrease in strength and an increase in shrinkage [19]. Huang et al. [11] completely replaced the lime in AAC production with skarn-type copper tailings and blast furnace slag. Using skarn-type copper tailings, furnace slag, cement clinker, and gypsum as the raw material composition, AAC with a compressive strength of 4.0 MPa was produced.

A review of the literature reveals that, in recent years, very limited studies [11,19] have been conducted on replacing lime in AAC production with waste materials. The objective of this study was to investigate the feasibility of using dry waste originating from the cleaning of flue gases from the combustion of industrial wastes in the production of autoclaved concrete. Dry waste originating from the cleaning of flue gases from the combustion of industrial wastes (hereinafter referred to as “waste”) in the waste-to-energy process can be considered as an alternative binder to lime. To the authors’ knowledge, the substitution of such a waste for lime as a calcareous source has not been investigated. Among pollutants present in flue gases formed during the waste-to-energy conversion, there are acidic gases such as hydrogen chloride or sulfur dioxide which are of major concern [22]. Various techniques may be used in order to separate those compounds [23]. Detailed information on the formation of such products may be found in [24,25].

The solid waste investigated in the present study is a product of the treatment of flue gases with calcium hydroxide slurry. The product is a fine powder containing unreacted calcium hydroxide (Ca(OH)_2_) in the form of portlandite, calcium chloride hydrate (CaClOH), halite (NaCl), and calcium carbonate (CaCO_3_) in the form of calcite. Waste is formed as a result of the reaction between flue gases containing acidic compounds (mainly hydrogen chloride) and lime slurry in an industrial waste incinerating plant. The process results in a solid dry residue in the form of a fine, dusty white powder. It is collected in bag filters made of PTFE, which are periodically cleaned, and dust is collected and then further processed/stored. To ensure the proper degree of absorption of harmful gases, excess of sorbent is needed, which is left unreacted and is, thus, a potential raw material for further utilization. What should be underlined is that such waste contains decarbonized calcium oxide, which is of great importance from the point of view of sustainable development.

Introduction of the investigated waste into AAC technology may be beneficial from both economic and environmental points of view. The reduction in greenhouse gases has become a priority issue in various industries, including the building materials industry [26,27]. Similarly to cement production, industrial lime production results in carbon dioxide emissions [28]. Emissions of CO_2_ in the lime industry mainly come from the calcination of limestone into calcium oxide. Waste as a calcareous material is a source of calcium oxide, thus decreasing the carbon footprint of the process. In addition, replacing part of lime with calcareous waste allows decreasing the amount of waste disposal in landfills. This is especially important since the investigated waste contains a very large fraction of soluble compounds and is, thus, very difficult to store.

The activity of waste used in present study was improved by calcination, and calcined waste was also used in the investigation. The bulk density, compressive strength, thermal conductivity, pore size distribution, phase composition, and microstructure of AAC containing waste were investigated.

## 2. Materials and Methods

### 2.1. Raw Materials

The raw materials for AAC sample preparation were cement, lime, quartz sand, and aluminum powder. The quartz sand was ground in a laboratory ball mill to a specific Blaine surface area of 2000 cm^2^/g [29]. The ordinary Portland cement (OPC) CEM I 42.5R conforming to the EN 197 standard was used as the cementitious material. Pure p.a. calcium carbonate delivered by POCH (Gliwice, Poland) was calcined at 1000 °C for 2 h to obtain a CaO source. Aluminum powder with a water coverage of 5500 cm^2^/g according to the procedure described in PN-77/H-04949 [30] standard was used as a pore-generating agent.

In the present study, solid waste from flue gas cleaning in an industrial waste incinerating plant was used as received, as well as after thermal treatment. The chemical composition of the raw waste is presented in Table 1. The waste was classified as 19 01 07* “solid wastes from gas treatment” according to EUROSTAT Guidance on EWC-Stat Waste Categories [31]. Thermal treatment of raw waste was applied in order to increase the reactivity of the material. Calcium hydroxide present in raw waste albeit reactive is not as reactive as calcium oxide. The material was heated for 1 h at 550 °C. This temperature was chosen in order to allow calcium hydroxide to decompose [32]. On the other hand, to obtain a material of good reactivity, the temperature should not be too high, since thermal treatment of calcium oxide at elevated temperatures may cause sintering, recrystallization, and grain growth, which negatively influence reactivity [33]. The temperature was also chosen to ensure that chlorine was not released from the sample during thermal treatment. This was confirmed by the results of the thermal analysis investigations presented in Section 3.1.

### 2.2. Mix Proportions

For the preparation of reference mix OPC, burnt lime, quartz sand, aluminum powder, and water were used as raw materials. The raw waste and calcined waste were used to replace some of the lime. The dosage of waste was calculated on the basis of calcium oxide mass content in the form of calcium hydroxide. Burnt lime used as a raw material for AAC preparation was replaced at a level of 10% by weight with the calcium hydroxide/calcium oxide from raw waste/calcined waste, respectively. Calcium oxide in the form of calcium hydroxide was taken into account during calculations. The mix proportions are summarized in Table 2. In the experiment, the AAC samples with raw waste and calcined waste were coded as R-AAC and C-AAC, respectively.

The AAC samples were prepared as follows: the weighed OPC, lime, and ground quartz sand were first mixed for 2 min. Then, the water was added and mixed for another 30 s. Finally, the aluminum powder suspension (prepared from aluminum powder and about 25 mL of mixing water taken prior to mixing) was added. The water/solid (*w*/*s*) ratio was constant for all the mixes. It was found that the introduction of the waste did not significantly change the workability of the mixes. The slurry was poured into preheated steel molds with dimensions of 100 mm × 100 mm × 100 mm and cured at the temperature of 60 °C for 2 h. After demolding, the samples were autoclaved at a temperature 180 °C for 8 h in a laboratory autoclave.

### 2.3. Testing Procedure

X-ray fluorescence spectrometry (XRF) was performed to obtain the chemical composition of waste. XRF analysis was performed using a PANalytical WDXRF Axios mAX spectrophotometer. The morphology of raw waste was characterized using a JEOL JEM-1011 transmission electron microscope. Thermal measurements (DTA/TG/MS analysis) were performed with a Netzsch STA 449 F3 Jupiter apparatus. The rate of heating was 10 °C/min. Analyses were performed in an inert atmosphere of helium (50 cm^3^/min). The composition of gases evolved during the thermal analysis of the sample was determined using a quadrupole mass spectrometer QMS 403 D Aëolos^®^. The Blaine specific surface area of quartz sand was determined according to EN 196-6 [29]. X-ray diffraction (XRD) was used to investigate phase composition of AAC samples. The phase compositions of the samples were examined using a Philips X-ray diffractometer X’pert system with monochromatic CuKa radiation. Pieces of material from the inside of the cube were taken, dried in an oven at 60 °C, and then ground with a mortar and pestle until the whole sample passed through a 0.063 mm sieve. The microstructure of samples was observed using a low-vacuum FEI NanoSEM 200 scanning electron microscope. Freshly broken fractured surfaces were sputtered with a thin carbon layer in order to avoid the charging of samples during observations. The bulk density was determined according to EN 772-13 [34]. The compressive strength test was conducted according to EN 772-1 [35]. An ISOMET 2104 (Applied Precision, Ltd., Bratislava, Slovakia) heat transfer analyzer was used to measure the thermal conductivity of the samples [36].

## 3. Results

### 3.1. Characterization of Waste

**Figure 1:**
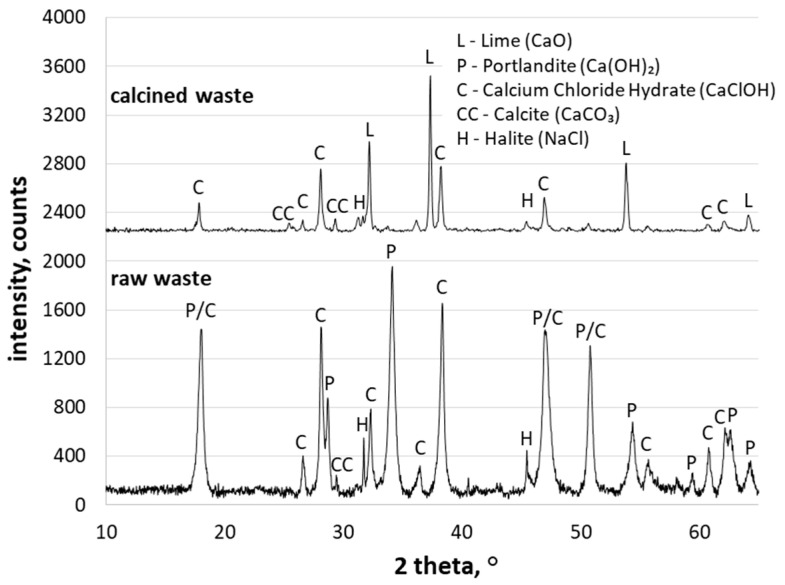
XRD patterns of raw and calcined waste.

**Figure 2:**
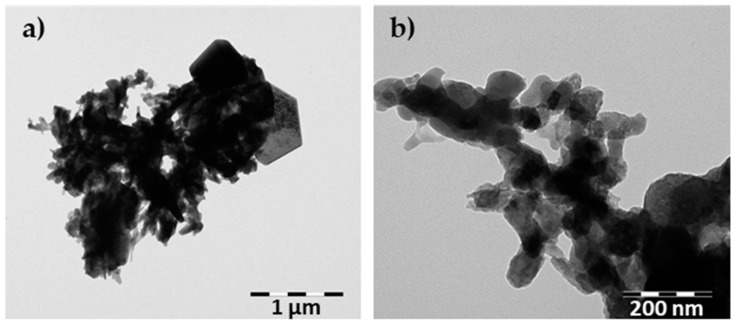
TEM microphotograph of raw waste: (**a**) hexagonal plate of portlandite together with fine grains of other compounds; (**b**) detailed view of fine grains of the waste.

Table 1 presents the chemical composition of the raw waste used in the experiments. As can be seen, the principal chemical compound of the waste was CaO, allowing its use as a calcareous material in AAC production. XRD analysis was conducted to compare changes in the phase composition of the waste before and after calcination. Figure 1 shows the XRD patterns of raw waste and after calcination for 1 h at 550 °C. It can be seen that the main crystalline phases were calcium hydroxide (Ca(OH)_2_) in the form of portlandite, calcium chloride hydrate (CaClOH), halite (NaCl), and calcium carbonate (CaCO_3_) in the form of calcite. As expected, portlandite was converted to lime. The mineral composition of wastes also allowed their consideration as alternative calcareous resources to reduce the consumption of lime. Figure 2 presents TEM observations of raw waste. The microphotograph of the raw waste shows the hexagonal plates of portlandite and irregular shapes of fine grains.

**Figure 3:**
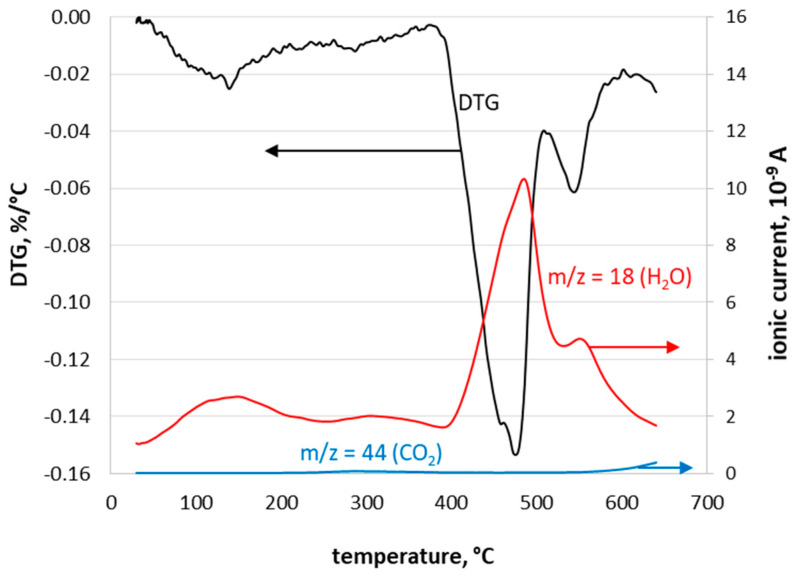
Differential gravimetric curve (DTG) of waste together with MS.

The thermal treatment of investigated waste at 550 °C did not cause any chlorine emissions during the process. Results of Dal Pozzo et al. [25] showed that chlorine starts to evolve from the sample at about 1000 °C. The examination performed for waste used in the present study showed that, within the range of temperatures applied, no chlorine evolved from the material during its thermal treatment. In Figure 3, results of thermal analysis of the sample are presented. The mass changes observed on DTG curves were caused by water release, as confirmed by the MS analysis (*m*/*z* = 18) of evolved gases. The massive loss of water with a maximum at about 490 °C was associated with calcium hydroxide decomposition [32]. No signal for *m*/*z* 36 or 37 was noted during the tests. This means that, under applied conditions, chlorine was stable in the sample.

### 3.2. Influence of Calcareous Waste on the Properties of AAC

#### 3.2.1. Bulk Density

The bulk density of AAC is related to its physical properties, especially its mechanical strength and thermal insulation. The effect of the addition of waste on the bulk density of AAC is shown in Table 3.

**Figure 4:**
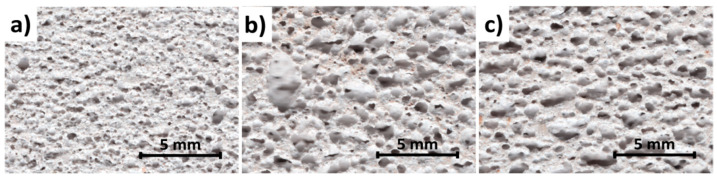
The porous microstructure of the investigated AAC samples: (**a**) reference sample; (**b**) R-AAC; (**c**) C-AAC.

The reference AAC, which was produced without waste, had a bulk density of 703 kg/m^3^. The bulk densities of the R-AAC and C-AAC samples were slightly lower than the bulk density of the reference sample. The introduction of waste into the AAC mixture, in both calcined and raw form, did not cause significant changes in the properties of the fresh mixture. It was observed that samples with waste achieved a higher final height of fresh mass compared to the reference samples, which was manifested by the lower density of concrete containing waste. The process underlying the increase in AAC mass is influenced by a number of factors, including the quality of the used raw materials, especially lime and aluminum powder. Moreover, in some cases, the process underlying the increase in AAC mass can be significantly affected by the introduced wastes [10,17,37]. Figure 4 shows the pore structure of AAC samples. As can be seen, the pores of the R-AAC and C-AAC samples were significantly coarser than the pores in the reference sample. The significant difference in pore microstructure observed between samples led to the decrease in bulk density.

#### 3.2.2. Compressive Strength

The changes in the mechanical properties of investigated concretes are given in Table 4.

**Figure 5:**
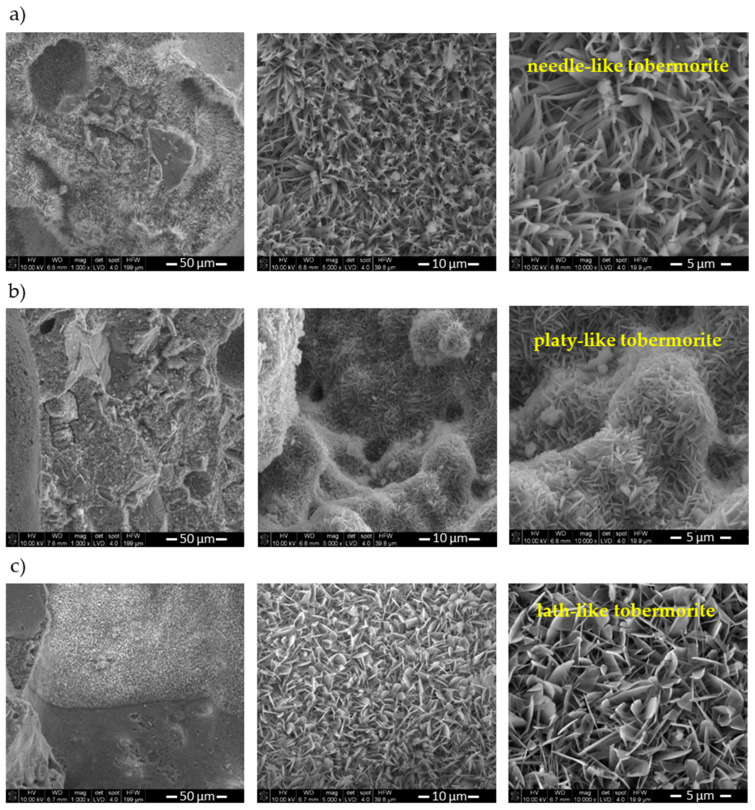
SEM microphotograph of AAC samples: (**a**) reference sample; (**b**) R-AAC; (**c**) C-AAC.

As shown in Table 4, the strength of the AAC samples increased as the bulk density decreased (Table 3). The replacement of lime by waste resulted in a significant increase in compressive strength. In the case of concrete with raw waste, the increase in strength was 50%, while, in the case of calcined waste, the increase was 31%. It is worth highlighting that these increases took place despite the slight decrease in concrete density by approximately 5%. According to [38], porosity has a significant influence on the compressive strength of AAC, and the reduction in bulk density and the related increase in porosity generally cause a reduction in strength. However, the increase in strength could not be related to the pore structure (the size, shape, and the distribution) due to the fact that samples with waste, characterized by a lower bulk density and bigger pore size, had a higher value of compressive strength. The enhancement of mechanical properties may be attributed to the microstructure of the AAC matrix. As for many materials, the microstructure of the AAC matrix has a great influence on its properties, particularly compressive strength and thermal conductivity, since the porosity of AAC is between 60% and 80% [38]. The microstructure of AAC also depends on the type and number of phases present within the matrix, the rate of hydration, type of reaction products formed, and their distribution in the AAC matrix [38]. These features strongly depend on the chemical composition of the raw mix and the course of hydrothermal treatment. Therefore, the compressive strength of AAC is governed not only by the porosity and distribution of pores in the AAC matrix but also by the type, amount, and microstructure of hydration products. In this study, the increase in the compressive strength of the AAC specimens produced with waste could be attributed to differences in the microstructure of the AAC matrix and alteration of the 1.1 nm tobermorite morphology. SEM images illustrate three different morphologies of 1.1 nm tobermorite: the needle-like 1.1 nm tobermorite crystals in the control sample (Figure 5a), plate-like 1.1 nm tobermorite crystals in the R-AAC sample (Figure 5b), and lath-like in the C-AAC sample (Figure 5c). It is worth noting that, as the lime was partially replaced by waste, the AAC microstructure became denser (Figure 5b,c). The microstructure of the hydration product (1.1 nm tobermorite) was found to be one of the major factors affecting strength development. The dense and compact microstructure of AAC with waste resulted in an increase in strength despite the increase in the size of the macropores generated by the evolution of hydrogen (Figure 4). These observations are consistent with some previous studies [37,39,40], where the formation of C–S–H phases with different microstructure and morphology was observed to have a significant effect on the compressive strength of AAC.

Moreover, the results obtained indicate that the compressive strength of the AAC samples was more affected by the morphology of the hydration product and related dense microstructure than the pore size distribution. It can be concluded that the dense microstructure of AAC was able to compensate for the loss of strength associated with the increase in macropore size distribution.

#### 3.2.3. Thermal Conductivity

The thermal conductivity, one of the most important physical properties of AAC, is widely considered by researchers [41,42,43,44,45,46,47]. Many factors may affect the thermal conductivity coefficient of AAC. It is well known that the thermal conductivity of AAC is governed by the porosity, the pore size distribution, and the thermal conductivity of individual components of the material [38]. Table 5 illustrates the impact of waste as a partial replacement of lime on the thermal conductivity of AAC.

The thermal conductivity of the samples with waste (R-AAC, C-AAC) was slightly higher than the reference value, and the values were found to be not strongly dependent on the bulk density. The lowest thermal conductivity value was observed in the control AAC. It was found to be 0.148 W/(m∙K). Partial replacement of lime with raw and calcined waste increased the thermal conductivity by 12% and 14%, respectively, with respect to the reference sample. This can be explained by differences in the porous microstructure of AAC. The increase in thermal conductivity of concrete that incorporated waste as a lime replacement could be attributed to the differences in the pore size distribution of samples (Figure 4). The pore size distribution is an important factor determining heat transport through porous materials. The tiny pores observed in the reference AAC (Figure 4a) indicated a lower thermal coefficient than the larger pores observed in samples containing waste (Figure 4b,c).

To achieve satisfactory thermal properties of AAC with waste, the pore size distribution should be optimized. The pore size distribution can be controlled during the technological process of AAC production. There are some methods for shaping the density and size of the pores in AAC technology, such as by changing the foam stabilizer content and stirring time [43] or fineness and dosage of aluminum powder [48]. Therefore, the factors related to foaming should be examined in the future.

### 3.3. X-ray Diffraction Analysis

**Figure 6:**
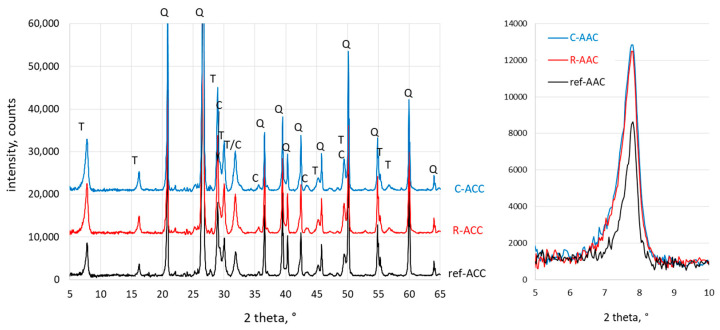
XRD pattern of AAC samples (T—1.1 nm tobermorite, Q—quartz, C—C–S–H phase). On the right, a comparison of the main tobermorite peaks for investigated concretes is plotted.

The phase composition of the AAC samples was examined using XRD. The diffraction patterns are presented in Figure 6.

The main constituents in autoclaved concrete were calcium silicate hydrates (1.1 nm tobermorite and the poorly crystalline C–S–H phase), which significantly affected the mechanical properties and durability of the ACC. According to literature data, the foreign ions introduced with wastes can affect the synthesis of calcium silicate hydrates [49,50,51,52,53,54]. For these reasons, the influence of investigated replacement materials on phase formation must be examined. Furthermore, the composition of the waste used is often a factor that influences the phase composition of aerated concrete [11,16]. For example, Peng et al. [16] produced autoclaved aerated concrete using graphite tailings as siliceous material. In the resulting material, hydration products typical for AAC were accompanied by muscovite originating from the unreacted raw materials [16]. In the present study, along with the waste, in addition to lime and calcium hydroxide, halite, calcite, and calcium chloride hydrate were introduced into the mixture of AAC. Some studies reported that the presence of calcite in starting materials may favor the formation of scawtite [19], which can reduce the compressive strength of AAC. As can be seen, the XRD patterns of the AAC samples were very similar. The introduction of calcareous waste as a lime partial replacement did not change the type of calcium silicate hydrates. XRD analysis (Figure 5) showed mainly the presence of characteristic binding phases of AAC: crystalline 1.1 nm tobermorite (2Ɵ = 7.81°, 1.13 nm) and poorly crystalline C–S–H phase (2Ɵ = 29.35°, 0.304 nm; 2Ɵ = 32.05°, 0.279 nm; 2Ɵ = 50.08°, 0.182 nm). Moreover, unreacted quartz (2Ɵ = 26.62°, 0.334 nm) was observed. However, the intensity of the 1.1 nm tobermorite peak at 1.13 nm (2Ɵ = 7.81°) was noticeably higher in AAC samples with waste compared to the reference sample. The calculated relative intensity of the 1.1 nm tobermorite peak for R-AA and C-AAC compared to the reference sample was 196% and 216%, respectively. Although the intensity of the peaks could not be used for direct quantification of phases, it allowed us to assume that the replacement of lime by calcareous waste promoted the formation of a 1.1 nm tobermorite phase. This observation corresponds well to the high compressive strength of samples with waste (as shown in Table 4). The XRD results indicate that foreign ions introduced with the waste influenced the synthesis of 1.1 nm tobermorite, which had a positive effect on the mechanical properties of AAC samples.

### 3.4. Microstructure Characterization

The SEM images of the AAC samples are shown in Figure 5. It can be seen that well-crystallized 1.1 nm tobermorite formed in all AAC samples. It can be seen that the replacement of lime with calcareous waste led to changes in the morphology of 1.1 nm tobermorite crystals. The SEM image illustrates the needle-like 1.1 nm tobermorite crystals in the reference sample (Figure 5a), plate-like 1.1 nm tobermorite crystals in sample with raw waste (Figure 5b), and lath-like in sample with calcined waste (Figure 5c). The literature review demonstrates that the addition of some industrial wastes can significantly change not only the properties of ACC but also the morphology of calcium silicate hydrates, especially 1.1 nm tobermorite [9,15,55]. In recent years, many studies investigated the influence of different types of industrial waste on the synthesis and morphology of 1.1 nm tobermorite [56,57,58,59]. Depending on the starting materials used for the synthesis, 1.1 nm tobermorite crystals have crystal habits that range from whiskers or flakes [60] to laths [61,62], grass-like [10], tabular [58], fibers [63], plates [64], or needles [57]. The microstructure of the ACC with waste was denser than that of the reference sample. The dense microstructure of AAC and the differences in the morphology of 1.1 nm tobermorite made a positive contribution to the high compressive strength of AAC with waste. Thus, this finding shows good agreement with the compressive strength and the XRD results for the AAC produced. The 1.1 nm tobermorite size in R-AAC was obviously smaller than in the reference sample. As a result, the R-AAC sample was characterized by the highest compressive strength, since strength commonly increases with a decrease in grain size.

## 4. Conclusions

Our investigation of the use of calcareous waste from flue gas cleaning as a partial replacement for lime in the production of ACC resulted in the following conclusions:

1. Waste can be potentially used as a substitution of lime in AAC technology. This allows reducing the emissions of CO_2_ related to the limestone calcination process.
2. It was found that waste affects the properties of AAC by altering the morphology of 1.1 nm tobermorite and pore structure of AAC.
3. The compressive strength was found to increase with waste addition. The SEM analysis showed that the addition of waste resulted in densely packed plate 1.1 nm tobermorite crystals. The densified microstructure improved the mechanical properties of AAC. Additionally, it was shown that dense microstructure could compensate for the loss of strength associated with the increase in pore size distribution. The role of chlorine-bearing compounds present in the waste should be the subject of further tests, since it is possible that they influence the strength gain of AAC.
4. The thermal conductivity was found to increase with waste addition. The main reason for the increase in thermal conductivity was the pore size distribution significantly influenced by the waste addition.
5. The replacement of lime by waste promoted the formation and changed the morphology of 1.1 nm tobermorite.

## Figures and Tables

**Table 1:** Chemical composition of the waste (wt.%).

Composition	SiO_2_	Al_2_O_3_	Fe_2_O_3_	Na_2_O	K_2_O	CaO	MgO	TiO_2_	SO_3_	P_2_O_5_	Cl	Br	F
	2.03	0.43	0.55	3.15	0.75	67.45	0.39	0.14	5.09	0.07	18.43	0.14	0.08

**Table 2:** Mix proportions of autoclaved aerated concrete (kg/m^3^).

Sample	Mix Proportion (kg)					
	OPC	Quartz Sand	Lime	Aluminum Powder	Waste	Water
Ref.	78	531	99	0.4	0	347
R-AAC	78	531	89	0.4	27	347
C-AAC	78	531	89	0.4	25	347

**Table 3:** The bulk density of AAC samples.

Sample	Bulk Density (kg/m^3^)	Relative Bulk Density (%)
ref. AAC	703	100
R-AAC	667	95
C-AAC	677	96

**Table 4:** Compressive strength of AAC samples.

Sample	Compressive Strength (MPa)	Relative Strength (%)
ref. AAC	2.6	100
R-AAC	3.9	150
C-AAC	3.4	131

**Table 5:** Thermal conductivity of AAC samples.

Sample	Thermal Conductivity (W/(m·K))	Relative Thermal Conductivity (%)
ref. AAC	0.148	100
R-AAC	0.165	112
C-AAC	0.168	114

## Data Availability

The data presented in this study are available on request from the corresponding author.
